# Waste Windshield-Derived Silicon/Carbon Nanocomposites as High-Performance Lithium-Ion Battery Anodes

**DOI:** 10.1038/s41598-018-19529-1

**Published:** 2018-01-17

**Authors:** Mingu Choi, Jae-Chan Kim, Dong-Wan Kim

**Affiliations:** School of Civil, Environmental and Architectural Engineering, Korea University, Seoul, 02841 Republic of Korea

## Abstract

Silicon has emerged as the most promising high-capacity material for lithium-ion batteries. Waste glass can be a potential low cost and environmentally benign silica resource enabling production of nanosized silicon at the industry level. Windshields are generally made of laminated glass comprising two separate glass bonded together with a layer of polyvinyl butyral sandwiched between them. Herein, silicon/carbon nanocomposites are fabricated from windshields for the first time via magnesiothermic reduction and facile carbonization process using both waste glass and polyvinyl butyral as silica and carbon sources, respectively. High purity reduced silicon has unique 3-dimensional nanostructure with large surface area. Furthermore, the incorporation of carbon in silicon enable to retain the composite anodes highly conductive and mechanically robust, thus providing enhanced cycle stability.

## Introduction

For the last decade, lithium-ion batteries (LIBs) have been the primary energy storage device due to their many applications such as in electric vehicles, smart devices, and commercial drones^1–3^. However, commercial LIBs can barely accommodate high-energy applications because of the limitations of the graphite-LiCoO_2_ system. The replacement of graphite-based anodes is one of top priorities for advanced high-energy LIBs. The research and development of anode materials has focused on improving existing materials such as Si, Sn, Ge, and transition metal oxides^4–8^. Among them, silicon anodes have excellent potential owing to their low operation voltage and high maximum theoretical capacity of 4200 mA h g^−1^ ^9^. The substantial specific and volumetric capacity of silicon anodes can satisfy the demands of next-generation LIBs. However, there are some challenges for the commercial utilization of silicon anodes, including the large volume expansion, structural collapse, and unstable solid-electrolyte interphase (SEI) layer during the alloying process. These phenomena result in capacity decrease, poor Coulombic efficiency, and poor cycling retention^10,11^.

Many approaches have been studied to overcome these concerns of silicon anodes, including structural control, nano-engineering, active material isolation, coating, and the formation of composites with graphene and carbon nanotubes^11–15^. Among these, nano-engineering is one of the best methods for preparing silicon anodes with a large contact area, functional space, and flexible structural design. However, the synthesis of silicon nanostructures requires toxic and/or expensive precursors. When considering the cost and environmental friendliness, some researchers have suggested using industrial waste or organic matter in nature such as rice husks and petrochemical waste as silicon sources instead of chemical precursors^16–19^. These eco-friendly precursors can reduce the precursor cost; however, the reduction of silica is a high-energy and high-cost process. Silica reduction is conducted at high temperatures of over 1000 °C because it is difficult to break the strong O-Si-O bonds in silica materials. However, magnesiothermic reduction can reduce silica at relatively low temperatures of approximately 650 °C^20^. Recently, Favors *et al*. reported a 3D nanostructured silicon through the magnesiothermic reduction of beach sand^16^. Also, Li *et al*. reported silicon/carbon composite anode materials using glass bottles as raw materials^17^.

In this study, we focused on waste glass because it is mainly composed of amorphous silica (64%), which is a suitable silicon source. Waste glass is typically collected from various industrial wastes, such as windows, displays, bottles, and glass products. The collected waste glass is recycled for use as raw materials or manufactured goods. Laminated glass from windshield end in landfill because of polyvinyl butyral (PVB) adhesive films between two glasses^21^. The waste PVB film leads to a laborious recycling process to separate the glass and PVB film. However, the organic PVB film can be an appropriate carbon source due to its 67.6% carbon content. In laminated glass derived from waste windshields, the glass and PVB film can be the silicon and the carbon precursors, respectively. To the best of our knowledge, this work represents the first synthesis of a silicon/carbon composite anode for LIBs using waste windshields as both silicon and carbon precursors.

Herein, we synthesized 3D silicon anodes using glass from waste windshields via magnesiothermic reduction and acid-treatment. To further improve the electrochemical properties, silicon/carbon composites are fabricated using PVB films from windshields via a simple carbonization process. The silicon/carbon composite electrodes demonstrated high capacity and long cycle life due to their unique nanostructures and inclusion of conductive carbon.

## Results and Discussion

**Figure 1:**
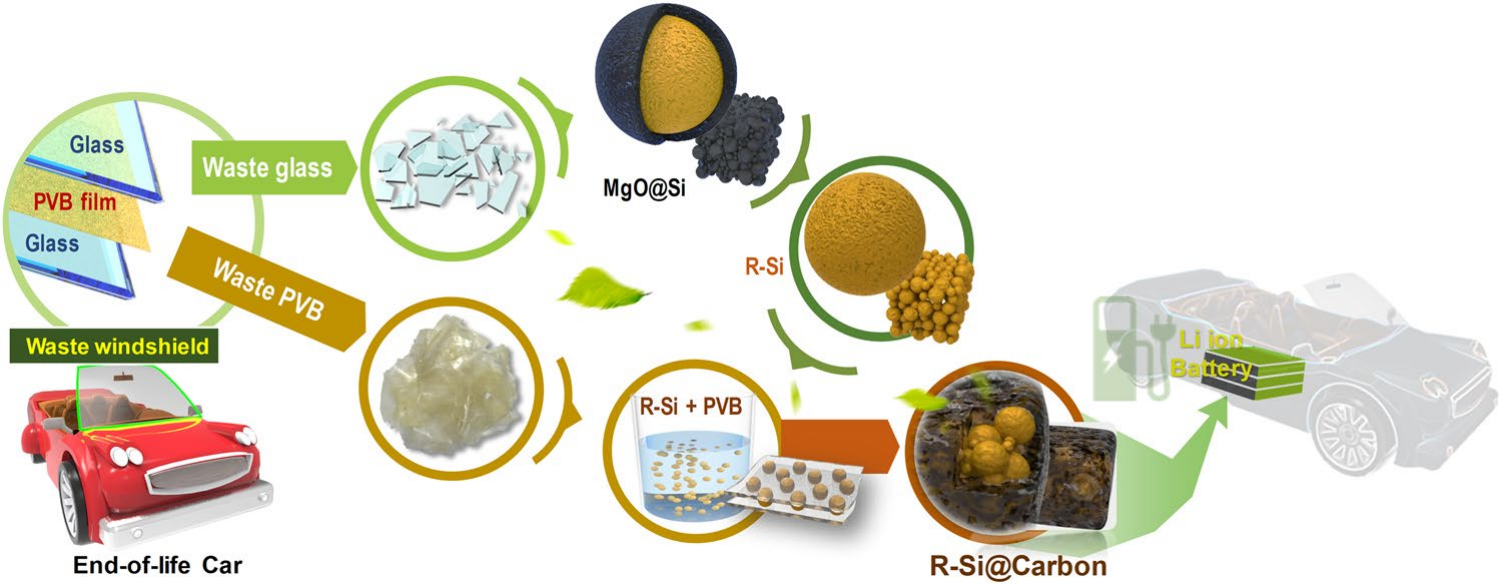
Schematic of the synthesis method of reduced silicon and silicon/carbon composites for lithium-ion batteries. All images, illustrations, and digital photographs were drawn and taken by J.-C. Kim.

Figure 1 depicts the two-step process used to fabricate the silicon/carbon composites using waste windshield. During the synthesis processes, reduced Si (R-Si) and R-Si/carbon composites are obtained; these samples are named R-Si@PVB*n* (*n* = 40 or 100) depending on the PVB/R-Si weight ratio.

**Figure 2:**
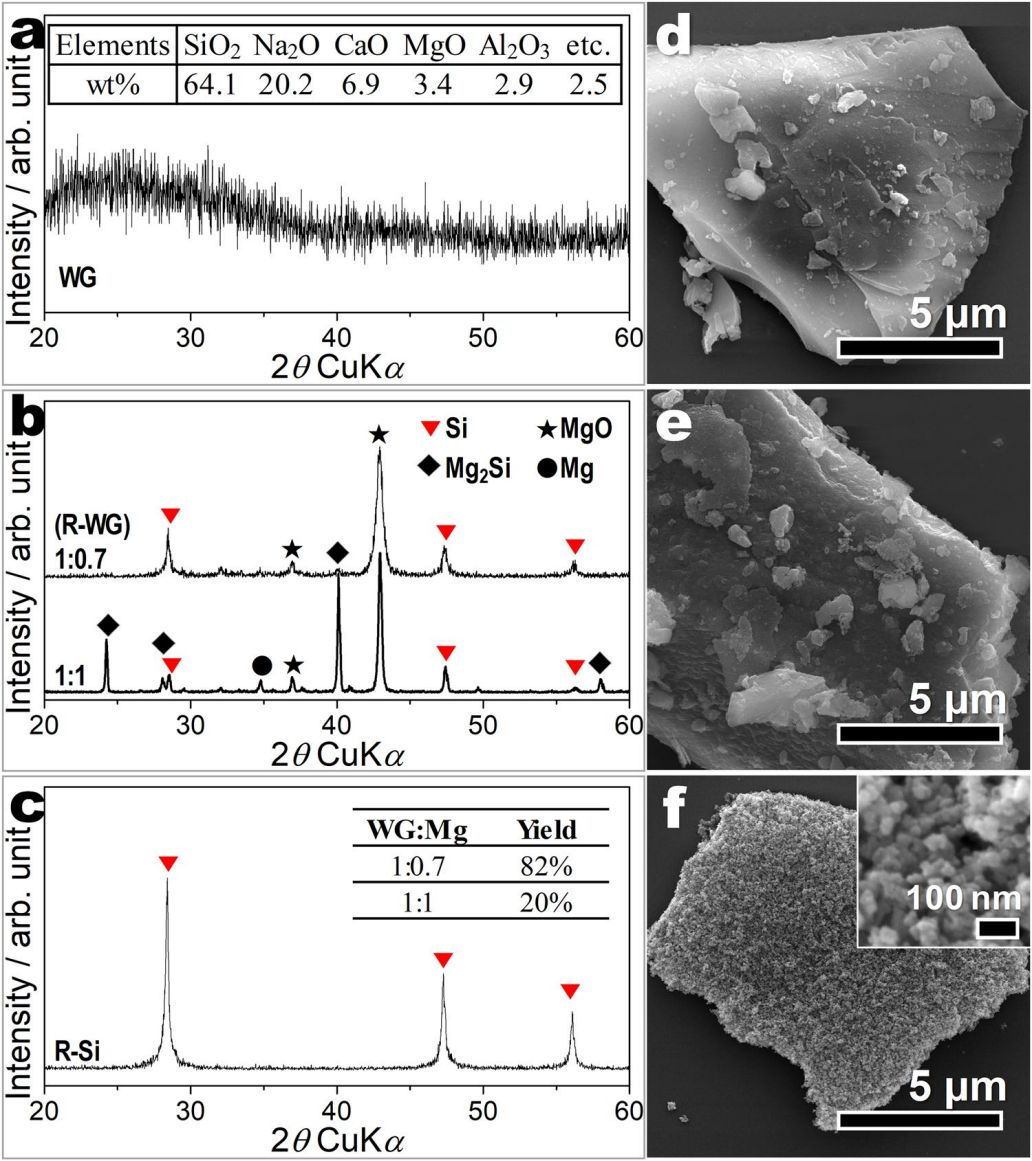
Morphology and structure analysis of waste glass, the reduced-waste glass (before acid etching process), and the reduced silicon (after acid-treatment process). XRD patterns of (**a**) WG (inset: The ICP-OES results of WG), (**b**) R-WG (WG:Mg = 1:0.7 and 1:1), and (c) R-Si. Inset in (**c**) shows the yield of R-Si. FE-SEM images of (**d**) WG, (**e**) R-WG (WG:Mg = 1:0.7), and (**f**) R-Si. Inset in (**f**) shows high-magnification FE-SEM images of R-Si.

The change in crystal structure is investigated based on the X-ray diffraction (XRD) patterns of waste glass (WG), reduced WG (R-WG) and R-Si (Fig. 2). Before the reduction process, the XRD pattern of WG is indicative of a typical amorphous structure without any crystalline phase (Fig. 2a). The silica content has been estimated to 64.1% based on inductively coupled plasma optical emission spectrometry (ICP-OES) results (Fig. 2a inset). The Fig. 2b shows the XRD patterns of R-WG as a function of WG: Mg ratios (1:1 and 1:0.7). XRD patterns in Fig. 2b can be indexed to Si (JCPDS #27–1402, marked with red reverse triangles), MgO (JCPDS #45–0946, marked with blue stars), and Mg_2_Si (JCPDS #35–0773, marked with black diamonds). During the magnesiothermic reduction, gaseous Mg reduces silica to silicon and MgO. However, side reactions also occur, producing magnesium byproducts such as Mg_2_Si and Mg_2_SiO_4_. This reaction proceeds according to the following equations^11,20^:

1 $$2\,{\rm{Mg}}({\rm{g}})+{{\rm{SiO}}}_{2}({\rm{s}})\to 2\,{\rm{MgO}}({\rm{s}})+{\rm{Si}}({\rm{s}})$$

2 $$2\,{\rm{Mg}}({\rm{g}})+{\rm{Si}}({\rm{s}})\to {{\rm{Mg}}}_{2}{\rm{Si}}({\rm{s}})$$

When the magnesiothermic reduction was carried out with relatively large amount of Mg (WG:Mg is 1:1), the intensity of Mg_2_Si peaks increased greatly (Fig. 2b). This indicated the enhanced side reaction (2), which caused a considerable consumption of reduced Si. Therefore, the yield of Si after acid-treatment increased to be 82% when the WG:Mg is 1:0.7 (Fig. 2c inset). More importantly, phase-pure Si with high crystallinity was obtained from WG.

The morphologies of WG and R-Si are identified by field emission scanning electronen microscope (FE-SEM). Figure 2d–f shows the typical FE-SEM images of WG and R-Si. As shown in Fig. 2d, WG has an irregular and bulky shape with a broad particle size distribution ranging from nanometer to several micrometers. The small particles are randomly distributed on the surfaces of the micro-sized particles. Figure 2e shows low-magnification FE-SEM image of R-WG (1:0.7), which have similar morphology with WG. After etching process, the bulky surface changed to a bumpy surface maintaining particle size (Fig. 2f). The high-magnification FE-SEM image in Fig. 2f inset shows that bulky WG is split into millions of nanoparticles. However, the overall micro-sized appearance of R-Si is maintained even after the reduction processes. Each nanoparticle has a mean diameter of approximately 30 nm. This 3D nanostructure R-Si has potential as an electrode material with good electrochemical performance because this unique structure offers a large contact area with electrolytes and reduces diffusion distance of lithium ions.

**Figure 3:**
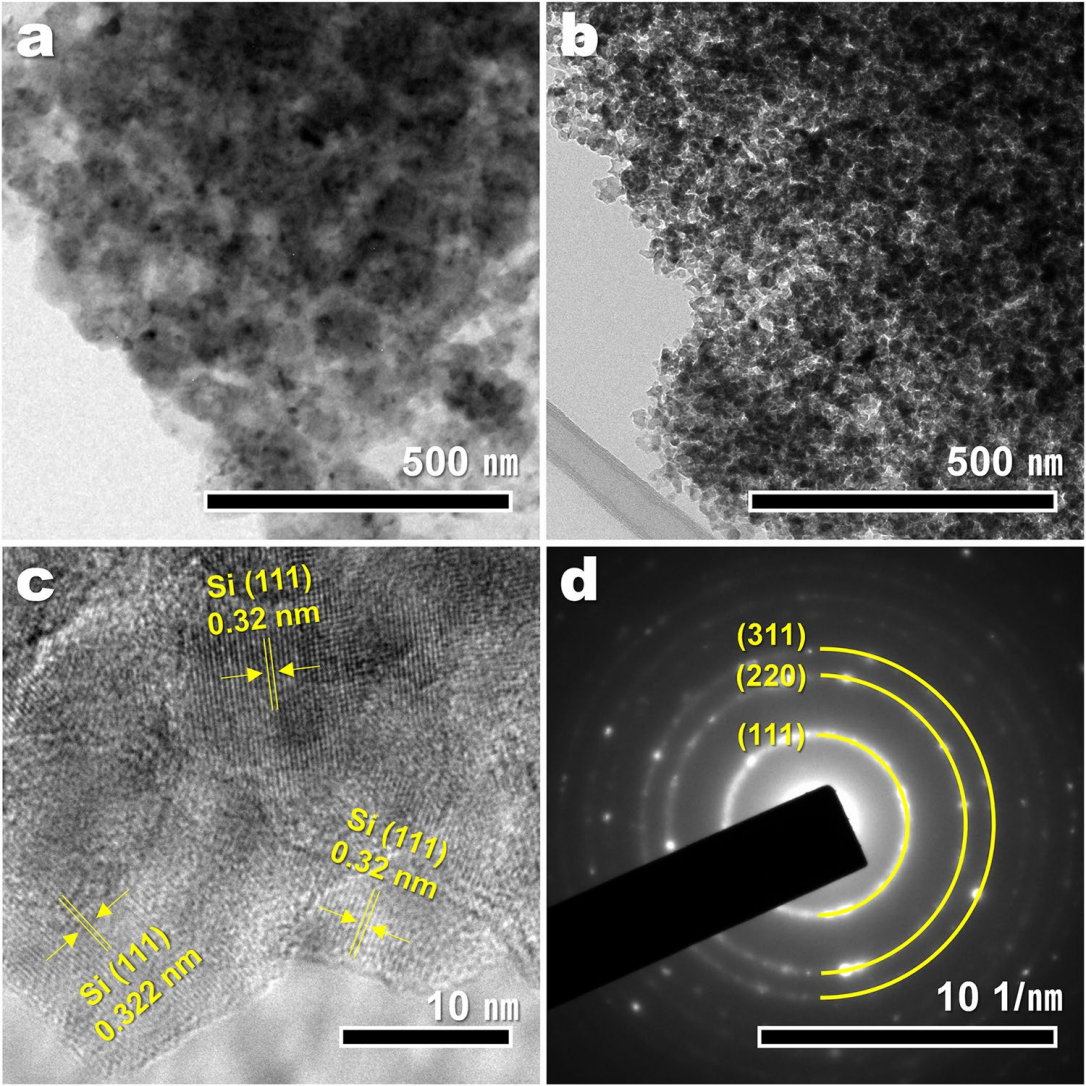
TEM characterization. TEM images of (**a**) R-WG and (**b**) R-Si. (**c**) HR-TEM image and (**d**) SAED pattern of R-Si.

Additional information about the 3D R-Si is obtained by transmission electron microscopy (TEM) analysis (Fig. 3). Figure 3a,b show low-magnification TEM images of R-WG and R-Si, respectively. In the TEM image of R-WG, the particles are heavily aggregated, and the boundary lines between particles are not clear. Bulky WG has already split into silicon nanoparticles in R-WG before the etching process. After the etching process, only ~30 nm of agglomerated nanoparticles substantially remain in R-Si. The matrix surrounding nanoparticles in Fig. 3a might be magnesium byproducts such as MgO and Mg_2_Si. Consequently, a highly porous structure is generated by etching the magnesium byproducts. Figure 3c presents the HR-TEM image of R-Si. The interplanar spacing of R-Si is measured to be 0.31 nm, which is consistent with the crystalline silicon (111) plane. Additionally, the selected area electron diffraction (SAED) pattern confirms the crystalline structure of silicon (Fig. 3d). The pattern exhibits a diffraction spots corresponding to the (111), (220), and (311) planes of silicon. The d-spacing or SAED patterns of excess silica or magnesium byproducts are not detected, indicating that WG is completely reduced to high-purity, single-crystalline silicon. The formation process of high-purity silicon with 3D nanostructure can be summarized in the following steps: (1) vaporized magnesium covers the WG surface; (2) the magnesium on the WG surface gradually diffuses into WG, and through the conversion reaction, silicon precipitates inside R-WG and magnesium byproducts are uniformly distributed both inside and outside R-WG; and (3) magnesium byproducts are completely removed by an acid-treatment. Finally, the silicon retained inside of R-WG maintains the unique 3D nanostructure.

**Figure 4:**
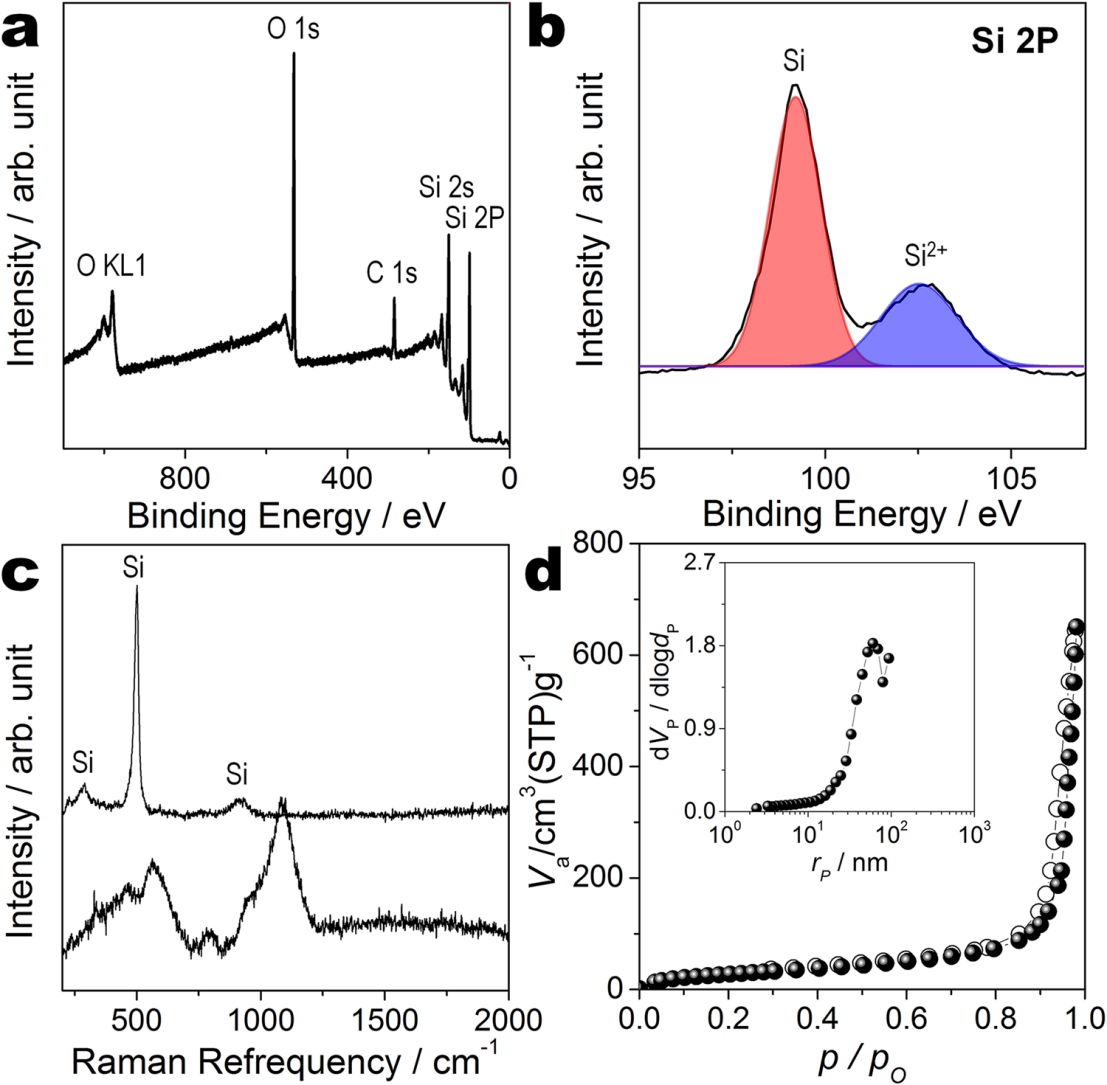
Component analysis and pore characterization of reduced silicon. XPS and Raman spectra of R-Si: (**a**) XPS survey spectrum, (**b**) XPS spectrum of Si 2p, (**c**) Raman spectra of R-Si (top) and WG (bottom), and (**d**) nitrogen adsorption–desorption isotherms of R-Si (inset: The BJH pore size distribution curve of R-Si).

For more accurate information on the crystal structure, the binding energy and Raman shift of R-Si are measured by X-ray photoelectron spectroscopy (XPS) and Raman spectroscopy. The elemental components of R-Si are identified from the XPS survey spectrum (Fig. 4a). Peaks are observed at around 979, 532, 284, 150, and 100 eV, corresponding to O KL1, O 1 s, C 1 s, Si 2 s, and Si 2p, respectively^14,22,23^. These results indicate that the metal oxides in WG and magnesium byproducts in R-WG are completely removed by the acid treatment. Figure 4b shows the narrow XPS spectrum of Si 2p. The peaks at 99–106 eV signify the electrovalence of the silicon atom. The binding energies of R-Si appear at 99 and 102 eV, which are attributed to Si-Si and Si-O bonds, respectively. The Si-Si peak height is more than three times higher than the Si-O peak height. R-Si includes the Si phase along with a trace of SiO phase. SiO may have originated from the native oxides on the surfaces of the silicon particles. Native silicon oxide is usually converted into silicon and oxygen atoms during the first few lithiation processes, and silicon atoms reversibly react as active materials^24–26^.

As shown in Fig. 4c, the Raman spectrum of WG reveals the typical soda-lime glass Raman shift^26,27^. The peaks are located at 560, 800, and 1080 cm^−1^, which are attributed to Si-O-Si symmetric stretching vibration, silicon motion in oxygen cage, and Si-O stretching vibration, respectively. The Raman spectrum of R-Si reveals three peaks at 290, 503, and 913 cm^−1^, which are indexed to crystalline silicon. The intense peak at 501 cm^−1^ is related to the transverse optical mode of silicon nanoparticles^23,27^. Amorphous Si-O-Si and Si-O bonds are broken to form crystalline silicon during the Mg-reduction process.

Furthermore, nitrogen adsorption-desorption measurements are conducted to determine the pore characteristics of R-Si (Fig. 4d). The Brunauer–Emmett–Teller (BET) surface area of R-Si is 108 m^2^ g^−1^. This high specific BET surface area results from the unique 3D structure of R-Si, which includes void space between nanoparticles. The inset of Fig. 4d shows the Barrett–Joyner–Halenda (BJH) curve of R-Si. The pore diameters are distributed from 30 to 80 nm, with a large total pore volume of 0.998 cm^3^ g^−1^. These results confirm the formation of uniform 3D porous nanostructures in R-Si.

**Figure 5:**
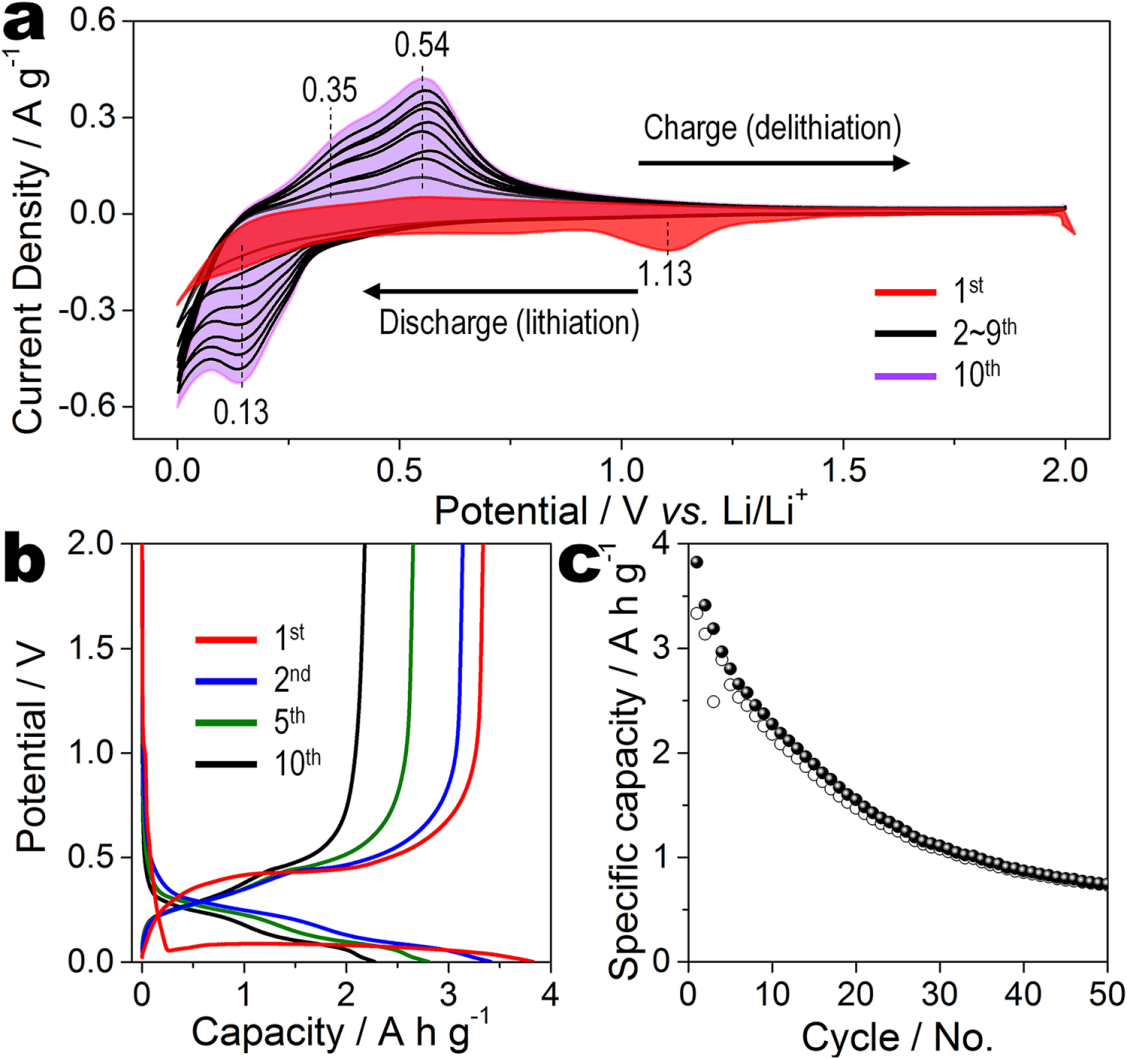
Electrochemical performance of reduced silicon. (**a**) Cyclic voltammetry curves of R-Si anode from the first to tenth cycles at a scan rate of 0.1 mV s^−1^ between 0.001 and 2.0 V. (**b**) Galvanostatic charge/discharge curves and (**c**) cycling performance of R-Si anode at a current density of 420 mA g^−1^; the current density for the first cycle is 210 mA g^−1^.

Figure 5 shows the electrochemical properties of R-Si electrodes. Cyclic voltammetry (CV) is conducted in the range of 0.01–2 V at a scan rate of 0.1 mV s^−1^ (Fig. 5a). During the first cathodic process, a prominent peak is observed at 1.13 V, which is attributed to the irreversible reaction between the R-Si electrodes and the electrolyte. A broad peak is also observed at 0.13 V, which is related to the alloy reaction of R-Si with lithium. In the first anodic process, two peaks are located at 0.35 and 0.54 V, which are the typical peaks of silicon anodes^28,29^. Both peaks correspond to the dealloying of Li_*x*_Si. The positions of the cathodic and anodic peaks are maintained during 10 lithiation/delithiation cycles, indicating the reversibility of the reaction between the R-Si electrode and lithium. However, the intensities of the redox peaks increase during subsequent cycles due to the activation of R-Si electrodes. This phenomenon can observe the previous literatures^22,28–31^.

To investigate the specific capacity and cycling retention, galvanostatic charge/discharge measurements are conducted in the potential range between 0.01 and 2 V with a current density of 420 mA h g^−1^. R-Si electrodes were cycled at a current density is 210 mA g^−1^ during 1^st^ cycle because the low current density significantly improved the initial Coulombic efficiency of R-Si (Figure S1). The discharge–charge curves of R-Si electrodes are shown in Fig. 5b. The first discharge curves have typical plateaus under 0.1 V and are attributed to the alloy reactions of silicon and lithium. These plateaus become broader after the second cycle because crystalline silicon becomes amorphous lithium silicide (a-Li_*x*_Si)^26^. The specific capacities of R-Si are 3825 and 3336 mA h g^−1^ at the first discharge and charge processes, respectively, and then the specific capacity nearly reaches the theoretical value of 4200 mA h g^−1^. However, it decays rapidly, possibly due to the repetitive volume expansion and shrinking of the R-Si electrode (Fig. 5c).

**Figure 6:**
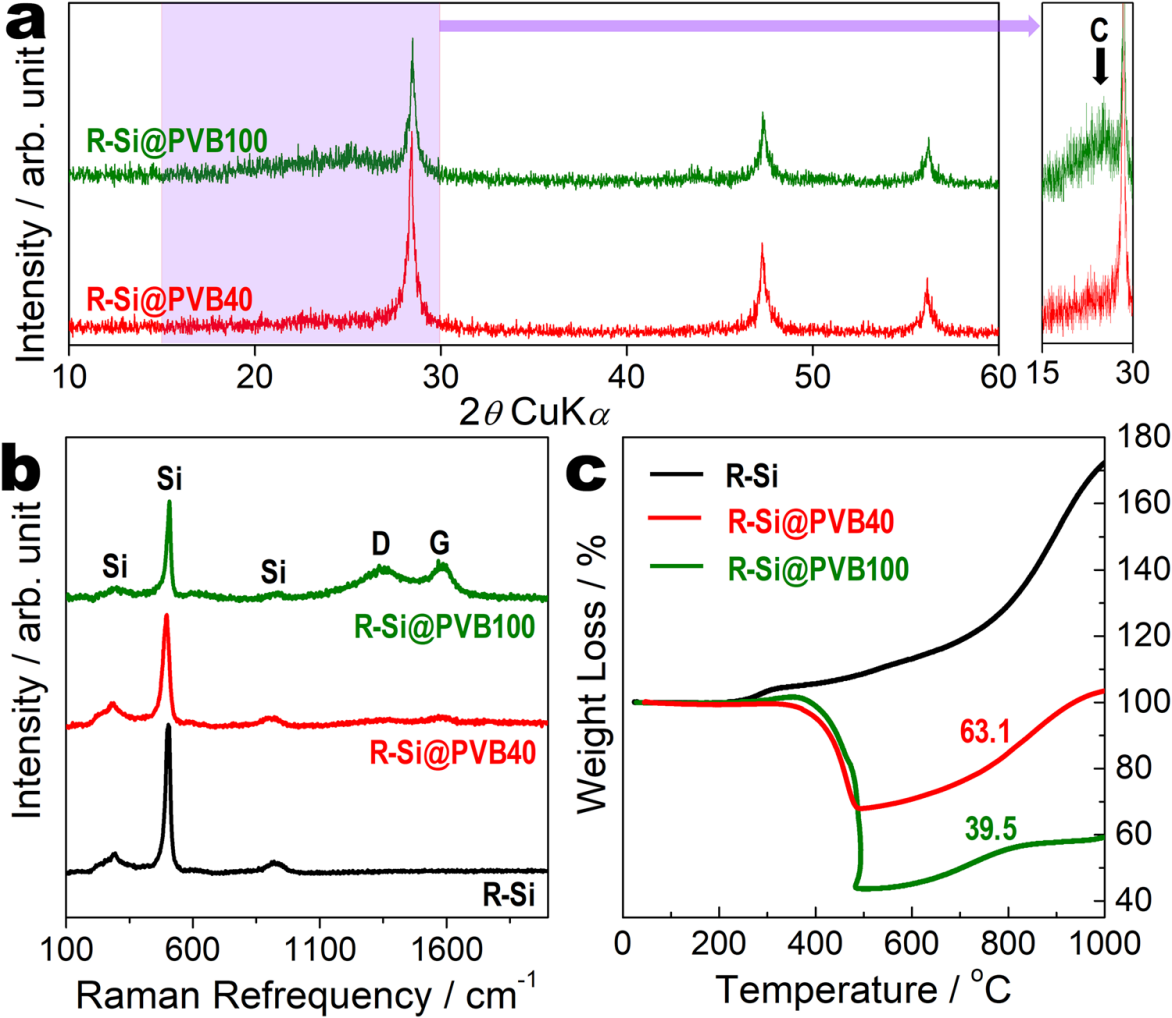
Structure and component analysis of the silicon/carbon composite. (**a**) XRD patterns of R-Si@PVB40 and R-Si@PVB100. (**b**) Raman spectra and (**c**) TGA curves of R-Si, R-Si@PVB40, and R-Si@PVB100.

To enhance the electrochemical performance, R-Si/carbon composite is also fabricated using waste PVB as the carbon source. R-Si is dispersed in waste PVB solution as waste PVB has high solubility in ethyl alcohol. R-Si particles are uniformly distributed in the waste PVB matrix after evaporating the solvent. XRD analyses are conducted to confirm the phases and crystal structures of R-Si@PVB40 and R-Si@PVB100 (Fig. 6a). The presence of carbon is clearly observed by the peak at ~25°, corresponding to the (200) plane of carbon, as magnified XRD pattern between 15 and 30° (Fig. 6a). R-Si@PVB100 had more carbon than R-Si@PVB40. The Raman spectra in Fig. 6b also demonstrate the inclusion of carbon. The Raman shift at 503 cm^−1^ is observed both before and after the carbonization process. However, the Raman shifts at 1351 and 1580 cm^−1^ are found only in the spectra of the R-Si@PVB40 and R-Si@PVB100 samples. The Raman peaks of R-Si@PVB40 are very weak. The *I*_D_/*I*_G_ values of R-Si@PVB40 and R-Si@PVB100 are measured to be 0.98 and 0.99, respectively. According to TGA analysis (Fig. 6c), the carbon contents of R-Si@PVB40 and R-Si@PVB100 are approximately 37% and 60%, respectively.

**Figure 7:**
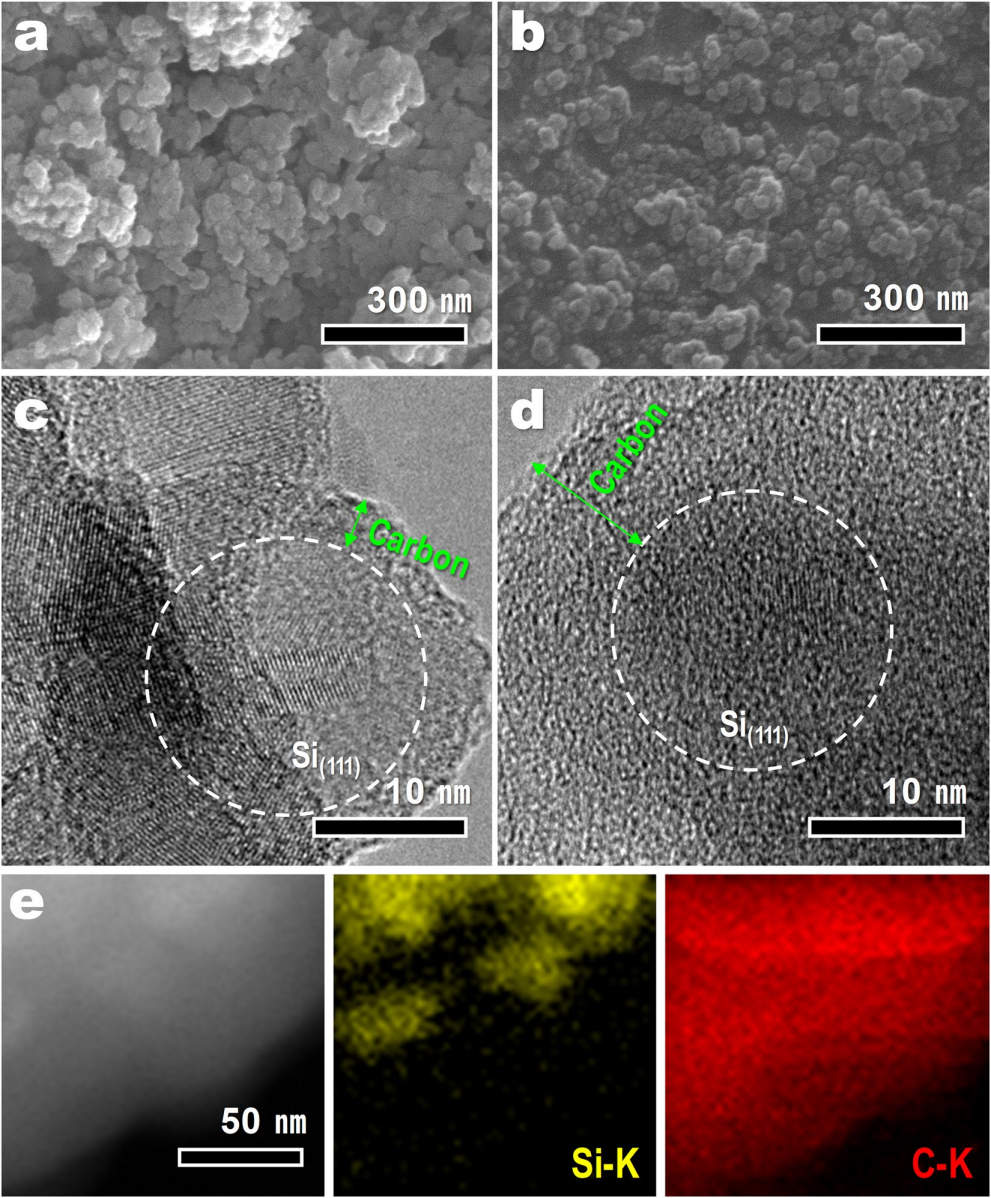
Morphology analysis of silicon/carbon composite. (**a**,**b**) Low-magnification FE-SEM images and (**c**,**d**) HR-TEM images of (**a**–**c**) R-Si@PVB40 and (**b**–**d**) R-Si@PVB100. (**e**) HAADF and EDS elemental mapping images of R-Si@PVB100.

The morphologies of R-Si@PVB40 and R-Si@PVB100 are demonstrated by FE-SEM and TEM images. As shown in Fig. 7a,b, the overall morphologies of R-Si@PVB40 and R-Si@PVB100 are similar to that of R-Si. However, the void space between the particles is significantly decreased compared to R-Si. The particle diameter of R-Si@PVB40 (~35 nm) is increased by ~5 nm compared to that of R-Si. Amorphous carbon with a thickness of a few nanometers encloses the silicon nanoparticles, accounting for the increase in particle size compared to R-Si. The HR-TEM image in Fig. 7c clearly shows the boundary between silicon and carbon. The FE-SEM image of R-Si@PVB100 (Fig. 7d) shows that silicon particles are embedded in the carbon matrix. The HR-TEM image (Fig. 7d) also reveals that Si exists only on the inside of R-Si@PVB100 at a distance of 10 nm from the carbon edge. The interplanar spacing of the white circles in Fig. 7c and d are indexed to the (111) plane of silicon. The high angled annual dark field (HAADF) image of R-Si@PVB100 was shown in Fig. 7e. The corresponding EDS elemental mapping clearly revealed the composition of R-Si@PVB100; Si-K signals (yellow dots) are concentrated on inner region of R-Si@PVB100 but C-K signals (red dots) covers the entire area of R-Si@PVB100. This result indicates that silicon nanoparticles were surrounded by conductive carbon matrix.

**Figure 8:**
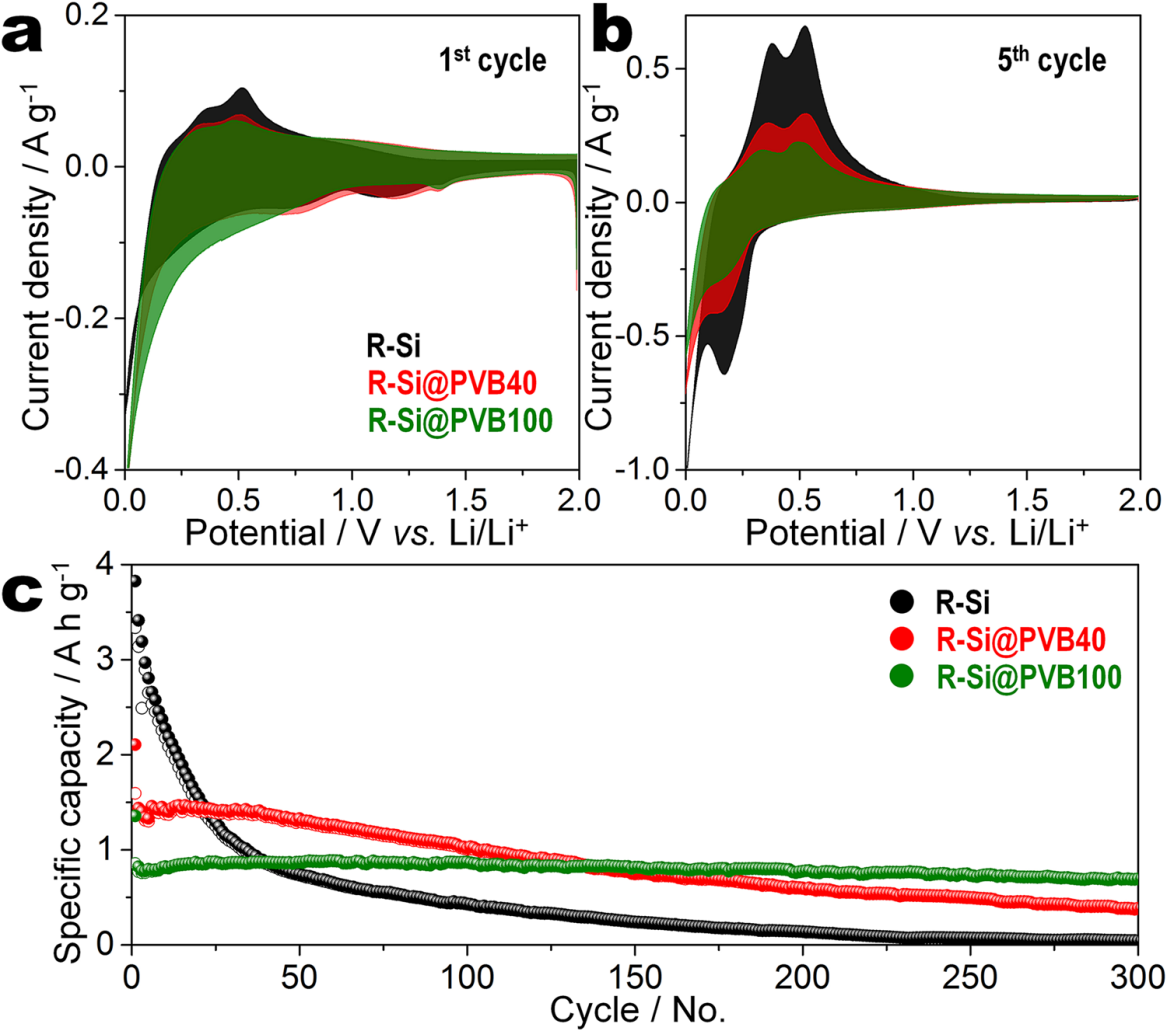
Electrochemical properties and long-term stability of silicon/carbon composite depending on PVB/silicon ratio. CV curves of R-Si, R-Si@PVB40, and R-Si@PVB100 for the (**a**) first and (**b**) fifth cycles. (**c**) Cycle performances of R-Si, R-Si@PVB40, and R-Si@PVB100 at a current density of 420 mA g^−1^; the current density for the first cycle is 210 mA g^−1^.

The electrochemical properties of R-Si@PVB*n* electrodes are demonstrated by CV measurements between 0.01 and 2.0 V at a rate of 0.1 mV s^−1^. Figure 8a and b show the CV curves of R-Si, R-Si@PVB40, and R-Si@PVB100 electrodes at the first and fifth cycles, respectively. In the first cathodic process, the peaks at 0.7 and 1.2 V are observed only for the R-Si and R-Si@PVB40 electrodes. These peaks indicate the irreversible decomposition of the electrolyte and the formation of an SEI layer. In the case of the R-Si@PVB100 electrode, a huge peak located under 0.1 V and a weak peak located at 1.4 V are observed. As shown in Fig. 8b, the redox peaks at the fifth cycle have the same locations as in the first cycle and the redox couples agree well with a typical silicon anode, corresponding to Li-Si alloying and dealloying processes. These CV results reveal that irreversible reactions are minimized in the first cycle when the R-Si@PVB100 electrode is used.

Figure S2a shows discharge/charge curves of R-Si, R-Si@PVB40, and R-Si@PVB100 electrodes at first cycle. The initial discharge capacity of R-Si, R-Si@PVB40 and 100 were 3825, 2105, and 1593 mA h g^−1^ with the Coulombic efficiency of 87.2, 75.6, and 63.1%, respectively. The discharge/charge curves of R-Si, R-Si@PVB40, and R-Si@PVB100 electrodes revealed a similar redox hysteresis, corresponding with the results of CV. During first discharge process, the plateaus were observed under 0.1 V indicating alloying reaction between silicon and lithium. The charge plateaus at 0.2 and 0.55 V revealed the decomposition reaction to silicon and lithium. The discharge/charge curves of R-Si@PVB40 and R-Si@PVB100 electrodes for 300 cycles were shown in Figure S2b and c. From second cycle, the discharge plateaus of R-Si@PVB40 and R-Si@PVB100 electrodes were observed at 0.4 V, which is corresponded to the results of CVs.

The capacity retentions of R-Si, R-Si@PVB40, and R-Si@PVB100 are investigated by galvanostatic cycling tests at a current density of 420 mA g^−1^ (Fig. 8c). Among the three samples, R-Si has the highest specific capacity (3825 mA h g^−1^) at the first cycle. However, the capacity of R-Si drops to 100 mA h g^−1^ during 200 cycles. The initial discharge and charge capacities of R-Si@PVB40 are 2105 and 1593 mA h g^−1^, respectively. The R-Si@PVB40 electrode maintains a capacity of ~1400 mA h g^−1^ until the 50th cycle and gradually decreases to 26% of the initial reversible capacity at the 300th cycle. The specific capacities of R-Si@PVB100 are 1356 and 856 mA h g^−1^ at the first discharge and charge processes, respectively. The capacity of Si@PVB100 is maintained at approximately 84% of the initial reversible capacity until the 300th cycle.

**Figure 9:**
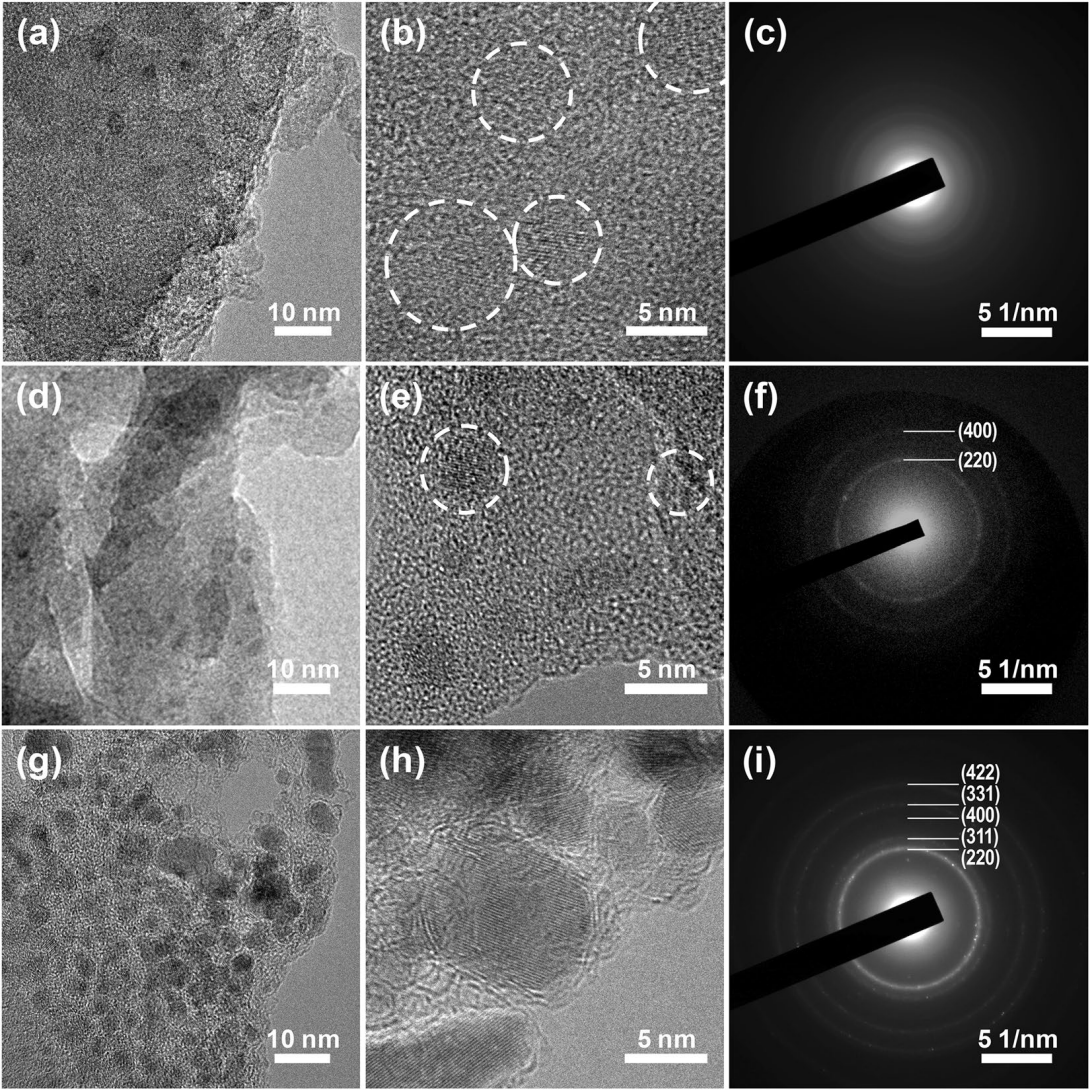
Morphology analysis of silicon/carbon composite anodes depending on PVB/silicon ratio. Low- and high-magnification FE-TEM image of (**a**,**b**) R-Si, (**d**,**e**) R-Si@PVB40, and (**g**,**h**) R-Si@PVB100 anodes at the 150^th^ charge state cycles. SAED patterns of (**c**) R-Si, (**f**) R-Si@PVB40, and (**i**) R-Si@PVB100 anodes at the 150^th^ charge state cycles.

Figure 9 shows the TEM images and SAED patterns of R-Si, R-Si@PVB40, and R-Si@PVB100 at 150^th^ charge state. Figure 9a, d, and g are the low magnification images of R-Si, R-Si@PVB40, and R-Si@PVB100, respectively. In the case of R-Si and R-Si@PVB40, the particles rarely exist in amorphous matrix. However, a lot of nanoparticles were observed in R-Si@PVB100 (Fig. 9g). The good cycling retention of R-Si@PVB100 electrodes can be attributed to the structure stability of R-Si@PVB100 during lithiation/delithiation process. Silicon particles can be clearly observed in the high resolution TEM images. (Fig. 9b,e and h). The particles in R-Si and R-Si@PVB40 have a low crystallity and they existed in amorphous matrix. However, high crystalline nanoparticles in R-Si@PVB40 were observed in Fig. 9h. The lattice fringes in R-Si, R-Si@PVB40, and R-Si@PVB100 were matched with cubic silicon (220) plane. The SAED patterns show crystalline nature of R-Si, R-Si@PVB40, and R-Si@PVB100 (Fig. 9c,f and i). The SAED patterns of R-Si and R-Si@PVB40 show faint ring patterns. However, R-Si@PVB100 have clear ring patterns and each spot in ring patterns exactly corresponded to cubic silicon (220), (311), (400), (331), and (422) planes. The R-Si@PVB100 includes enough carbon mitigating the volume change of silicon anodes upon cycling.

To understand electrochemical performances better, the electrochemical impedance spectroscopy (EIS) measurements were conducted to R-Si, R-Si@PVB40, and R-Si@PVB100 electrodes with a range 10 mHz to 100 kHz at 1^st^, 10^th^, 50^th^, and 100^th^ discharge/charge states. (Figure S3) The semicircles were observed in the high and middle frequency ranges and Warburg impedance region was observed in the low-frequency range. The semicircles of R-Si electrodes were smaller than carbon coated anodes until 10^th^ cycle. However, the semicircles of R-Si became larger and divided to two semicircles at 100^th^ cycle. These results represented the increase in charge transfer resistance, leading to rapid capacity drop of R-Si electrodes. In the case of R-Si@PVB40 and R-Si@PVB100, the semicircles were gradually shrank upon cycling. After 100 cycles, the semicircle of the R-Si@PVB40 and R-Si@PVB100 electrodes were much smaller than R-Si. These results indicated that the PVB-based carbon improved the electronic conductivity of R-Si@PVB40 and 100 electrodes, in comparison with the R-Si electrodes. The discharge and charge state had a similar EIS tendency upon cycling.

The electrode of R-Si@PVB100, in which R-Si is embedded in the carbon matrix, exhibits excellent cycling retention of 84% initial capacity at the 300th cycle. Figure S4a show galvanostatic discharge/charge curves and cyclability of pure PVB derived carbon electrodes. The specific discharge/charge capacity at the first cycle were 65 and 12 mA h g^−1^, respectively. The PVB derived carbon electrodes have small reversible capacity, ~10 mA h g^−1^ (Figure S4b) during further cycling. It is believed that the extra capacity from PVB derived carbon is negligible in the specific capacity of R-Si@PVB40 and R-Si@PVB100. Although the large carbon content decreases the specific capacity, the R-Si@PVB100 electrode exhibits the highest capacity after the 137th cycle. In addition, when we consider only the weight of silicon, the R-Si@PVB100 electrodes exhibit an excellent capacity of over 2000 mA h g^−1^ (Figure S5). The nanostructure and complexation with carbon in R-Si@PVB are the critical factors in the good electrochemical performance of R-Si@PVB electrodes. First, the 3D nanostructure of R-Si offers high surface area and prevents particle agglomeration. These features help retain good contact with the electrolyte. Additionally, the 3D nanostructure offers functional space to restrict the volume expansion of the silicon anode. Second, the uniform carbon coating provides high electrical conductivity and accommodates the volume change of silicon upon cycling. EIS results Moreover, the carbon in R-Si@PVB100 serves as a stable SEI layer and minimizes the irreversible reactions in the first cycle.

## Conclusion

We successfully synthesized 3D silicon/carbon composites via magnesiothermic and carbonization processes using inexpensive waste windshields. FE-SEM and TEM analyses revealed that the overall appearance of R-Si was similar to that of bulky WG; however, R-Si exhibited a porous 3D nanostructure composed of 30-nm nanoparticles. This unique 3D nanostructure originated from magnesium diffusion and reaction with silica precursors followed by etching through acid-treatment. R-Si had a high surface area of 108 m^2^ g^−1^ and a large total pore volume of 0.998 cm^3^ g^−1^. However, the specific capacities of R-Si electrodes decayed rapidly. To obtain better cycling retention, R-Si/carbon composites were fabricated using waste PVB film as the carbon resource. The R-Si@PVB40 and R-Si@PVB100 electrodes exhibited excellent cycling retention, compared with the R-Si electrodes. This cost-effective fabrication method, which produces a high-performance silicon/carbon composite anode material, is advantageous for its various potential use in recycling industry of waste windshields.

## Method

### Materials

Glass cullet and PVB films were collected from automotive windshield after crushing and separation processes. To remove the remaining dust and impurities, the waste glass was ground and treated by 5 M HNO_3_ aqueous solution at 70 °C for 96 h. Pre-treated waste glass powder (hereafter WG) was used as the silica precursor. The waste PVB films were used as the carbon precursor without any purification. The SiO_2_ content of WG was measured to be 64.1 wt% by inductively coupled plasma-optical emission spectroscopy (ICP-OES).

### Fabrication of reduced silicon

WG and magnesium powder (Hana AMT, > 99.5%) were mixed in a weight ratio of 1:0.7 (WG:Mg). The mixture was sealed on a stainless-steel ampoule in an argon-filled glovebox and heated in a tube furnace at 650 °C for 6 h at a heating rate of 1 °C/m under argon atmosphere. The products (Reduced-WG, R-WG) were immersed in 2 M HCl solution to remove the magnesium byproduct, and then, immersed in 5 wt% HF solution to remove the unreacted SiO_2_. The suspension was washed several times with deionized water and freeze-dried for 24 h. Finally, reduced silicon powders were obtained and termed R-Si.

### Fabrication of silicon/carbon composite

Waste PVB was dissolved in ethyl alcohol, and R-Si was uniformly dispersed in the PVB solution under vigorous stirring (PVB:R-Si = 40 or 100, wt/wt). This solution was dried overnight at 50 °C. The R-Si and PVB film were carbonized at 500 °C for 4 h at a heating rate of 50 °C/m under argon atmosphere. The final R-Si and carbon composites were termed R-Si/PVB40 and R-Si/PVB100 according to the PVB:R-Si ratio.

### Characterization of reduced silicon and silicon/carbon composite

The morphologies and compositions of the products were characterized by ICP-OES (OPTIMA 5300DV, PerkinElmer), high-resolution transmission electron microscopy (HR-TEM; JEM-2010, JEOL), selected area electron diffraction (SAED), and field-emission scanning electron microscopy (FE-SEM; Quanta 250 FEG, FEI). The crystal structure was characterized by powder X-ray diffraction (XRD; Ultima III, Rigaku), X-ray photoelectron spectroscopy (XPS; X-TOOL, ULVAC-PHI), and Raman spectroscopy (LabRam ARAMIS IR2, HORIBA JOBIN YVON). Thermogravimetric analysis (TGA) was performed on a thermal analyzer at a heating rate of 10 °C/m in air.

### Electrochemical performance of Li–O_2_ cells

R-Si and R-Si@PVB electrodes were prepared using 70% active materials, 15% super P, and 15% sodium alginate binder. The mixture slurry was coated onto a copper foil and dried in a vacuum oven at 100 °C for 4 h. The active loading mass of R-Si, R-Si@PVB40, and R-Si@PVB100 electrodes were between 1.5 and 2.0 mg cm^−2^. Swagelok-type half cells were assembled using R-Si and R-Si@PVB electrodes as working electrodes, lithium foils as the counter electrodes, and a liquid electrolyte of 1.0 M LiPF_6_ ethylene carbonate/propylene carbonate/diethyl carbonate (EC/PC/DEC = 1:1:2 v/v) containing 10 wt% fluoroethylene carbonate additive. The electrochemical properties of the electrodes were tested against Li from 0.001 to 2.0 V using a galvanostatic cycling tester (WBCS 3000, Wonatech, Korea) at varying current densities. Cyclic voltammetry (CV) was performed for 10 cycles at a scan rate of 0.1 mV s^−1^ over the same voltage range.

## Electronic supplementary material

Supplementary Information

## References

[1] Scrosati B, Hassoun J, Sun YK (2011). Lithium-ion batteries. A look into the future. Energy Environ. Sci..

[2] Armand M, Tarascon JM (2008). Building better batteries. Nature.

[3] Tarascon JM, Armand M (2001). Issues and challenges facing rechargeable lithium batteries. Nature.

[4] Kennedy T, Brandon M, Ryan KM (2016). Advances in the application of silicon and germanium nanowires for high-performance lithium-ion batteries. Adv. Mater..

[5] Lou XW, Li CM, Archer LA (2009). Designed synthesis of coaxial SnO_2_@carbon hollow nanospheres for highly reversible lithium storage. Adv. Mater..

[6] Lee GH, Lee S, Lee CW, Choi C, Kim DW (2016). Stable high-areal-capacity nanoarchitectured germanium anodes on three-dimensional current collectors for Li ion microbatteries. J. Mater. Chem. A.

[7] Wang B, Chen JS, Wu HB, Wang Z, Lou XW (2011). Quasiemulsion-templated formation of α-Fe_2_O_3_ hollow spheres with enhanced lithium storage properties. J. Am. Chem. Soc..

[8] Haregewoin AM, Wotango AS, Hwang BJ (2016). Electrolyte additives for lithium ion battery electrodes: progress and perspectives. Energy Environ. Sci..

[9] Piper DM (2016). Optimized silicon electrode architecture, interface, and microgeometry for next-generation lithium-ion batteries. Adv. Mater..

[10] Shi F (2016). Failure mechanisms of single-crystal silicon electrodes in lithium-ion batteries. Nat. Commun..

[11] Zhang R (2014). Highly reversible and large lithium storage in mesoporous Si/C nanocomposite anodes with silicon nanoparticles embedded in a carbon framework. Adv. Mater..

[12] Kim YM (2015). Titanium silicide coated porous silicon nanospheres as anode materials for lithium ion batteries. Electrochim. Acta.

[13] Casimir A (2016). Silicon-based anodes for lithium-ion batteries: Effectiveness ofmaterials synthesis and electrode preparation. Nano Energy.

[14] Li B, Li S, Wang B, Liu J (2015). From commercial sponge toward 3D graphene-silicon networks for superior lithium storage. Adv. Energy Mater..

[15] Agyeman DA, Song K, Lee GH, Park M, Kang YM (2016). Carbon-coated Si nanoparticles anchored between reduced graphene oxides as an extremely reversible anode material for high energy-density Li-ion battery. Adv. Energy Mater..

[16] Favors Z (2014). Scalable synthesis of nano-silicon from beach sand for long cycle life Li-ion batteries. Sci. Rep..

[17] Li C (2017). Silicon Derived from glass bottles as anode materials for lithium ion full cell batteries. Sci. Rep..

[18] Zhang YC (2016). Rice husk-derived hierarchical silicon/nitrogen-doped carbon/carbon nanotube spheres as low-cost and high-capacity anodes for lithium-ion batteries. Nano Energy.

[19] Kim SK (2016). One-step formation of silicon graphene composites from silicon sludge waste and graphene oxide via aerosol process for lithium ion batteries. Sci. Rep..

[20] Kim KH (2015). Complete magnesiothermic reduction reaction of vertically aligned mesoporous silica channels to form pure silicon nanoparticles. Sci. Rep..

[21] Tupy M, Mokrejs P, Merinska D, Svoboda P, Zvonicek J (2014). Windshield recycling focused on effective separation of PVB sheet. J. Appl. Polym. Sci..

[22] Li Q (2015). Mesoporous silicon/carbon hybrids with ordered pore channel retention and tunable carbon incorporated content as high performance anode materials for lithium-ion batteries. Energy.

[23] He D (2015). Fabrication of sandwich-structured Si nanoparticles-graphene nanocomposites for high-performance lithium-ion batteries. Electrochim. Acta.

[24] Yan N (2013). Hollow porous SiO_2_ nanocubes towards high-performance anodes for lithium-ion batteries. Sci. Rep..

[25] Zhang T (2007). Preparation and electrochemical properties of core-shell Si/SiO nanocomposite as anode material for lithium ion batteries. Electrochem. Commun..

[26] Yang J (2002). SiO_x_-based anodes for secondary lithium batteries. Solid State Ion..

[27] Deschamps T, Martinet C, Bruneel JL, Champagnon B (2011). Soda-lime silicate glass under hydrostatic pressure and indentation: a micro-Raman study. J. Phys.-Condens. Matter.

[28] Yoo S, Kim H, Kang B (2016). Characterizing local structure of SiO_x_ using confocal µ-Raman spectroscopy and its effects on electrochemical property. Electrochim. Acta.

[29] Wang W (2015). Monodisperse porous silicon spheres as anode materials for lithium ion batteries. Sci. Rep..

[30] Zhang Y (2015). Preparation of nanographite sheets supported Si nanoparticles by *in situ* reduction of fumed SiO_2_ with magnesium for lithium ion battery. J. Power Sources.

[31] Chen Y, Du N, Zhang H, Yang D (2015). Facile synthesis of uniform MWCNT@Si nanocomposites as high-performance anode materials for lithium-ion batteries. J. Alloy. Compd..

