# Supplementary material for: Waste Windshield-Derived Silicon/Carbon Nanocomposites as High-Performance Lithium-Ion Battery Anodes
Source: Sci Rep. 2018 Jan 17;8:960. doi: 10.1038/s41598-018-19529-1 (PMC5772056; doi:10.1038/s41598-018-19529-1)
Supplement: Supplementary file 1 — Supplementary Information [file 41598_2018_19529_MOESM1_ESM.pdf]

# Supplementary Information

## Waste Windshield-Derived Silicon/Carbon Nanocomposites as High-Performance Lithium-Ion Battery Anodes

Mingu Choi*, Jae-Chan Kim* & Dong-Wan Kim

School of Civil, Environmental and Architectural Engineering, Korea University, Seoul 02841, Republic of Korea

* These authors contributed equally to this work.

Correspondence and requests for materials should be addressed to D.W.K. (E-mail: dwkim1@korea.ac.kr)

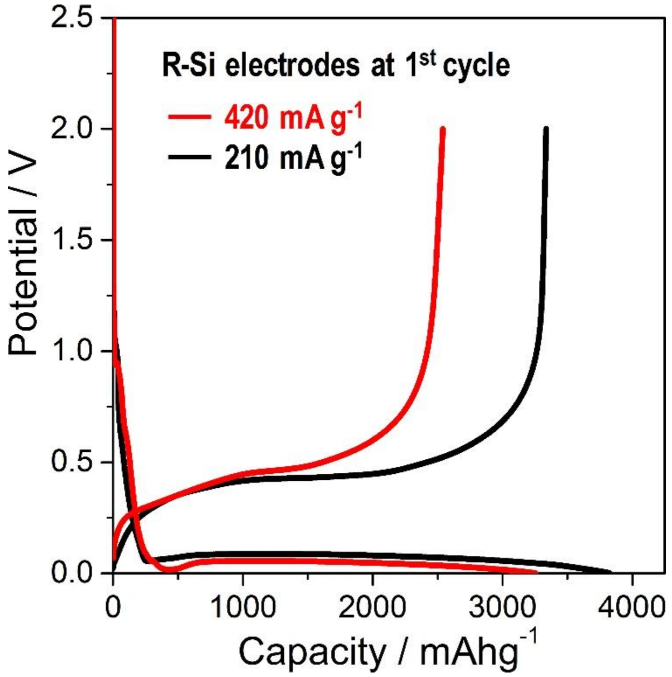


**Figure S1.** The discharge/charge curves of R-Si at the current density of 420 and 210 mA $g^{-1}$.

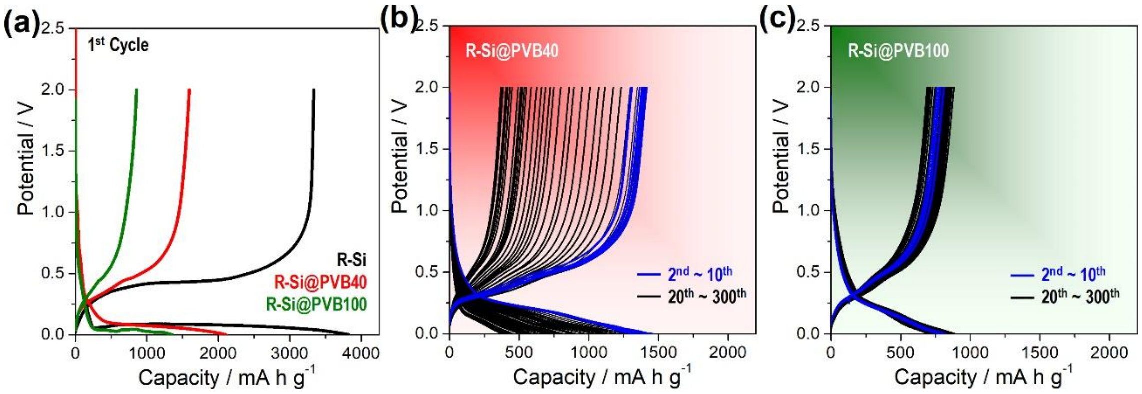


**Figure S2.** (a) The discharge/charge curves of R-Si, R-Si@PVB40 and R-Si@PVB100 electrodes at the first cycle. The discharge/charge curves of (b) R-Si@PVB40 and (c) R-Si@PVB100 electrodes for 300 cycles. Galvanostatic cycling tests operated between 0.01 and 2 V at a current density of 420 mA $g^{-1}$; the current density for the first cycle is 210 mA $g^{-1}$.

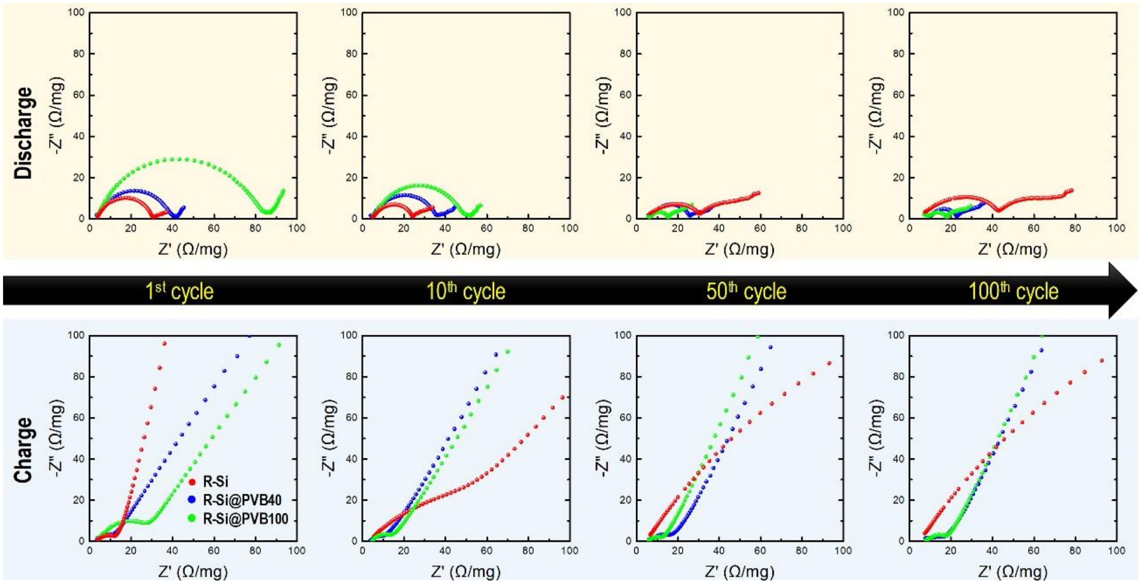


**Figure S3.** EIS measurements of R-Si, R-Si@PVB40, and R-Si@PVB100 electrodes at 1st, 10th, 50th, and 100th discharge/charge state.

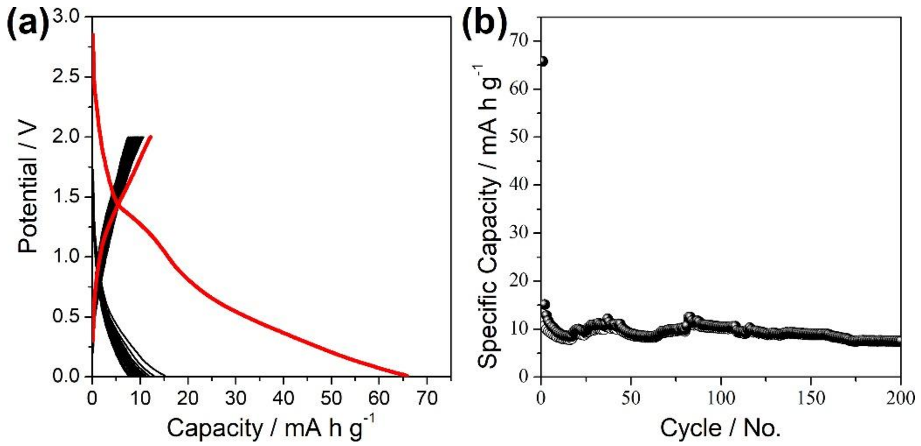


**Figure S4.** (a) The discharge/charge curves and (b) galvanostatic cycling graph of pure PVB derived carbon electrodes.

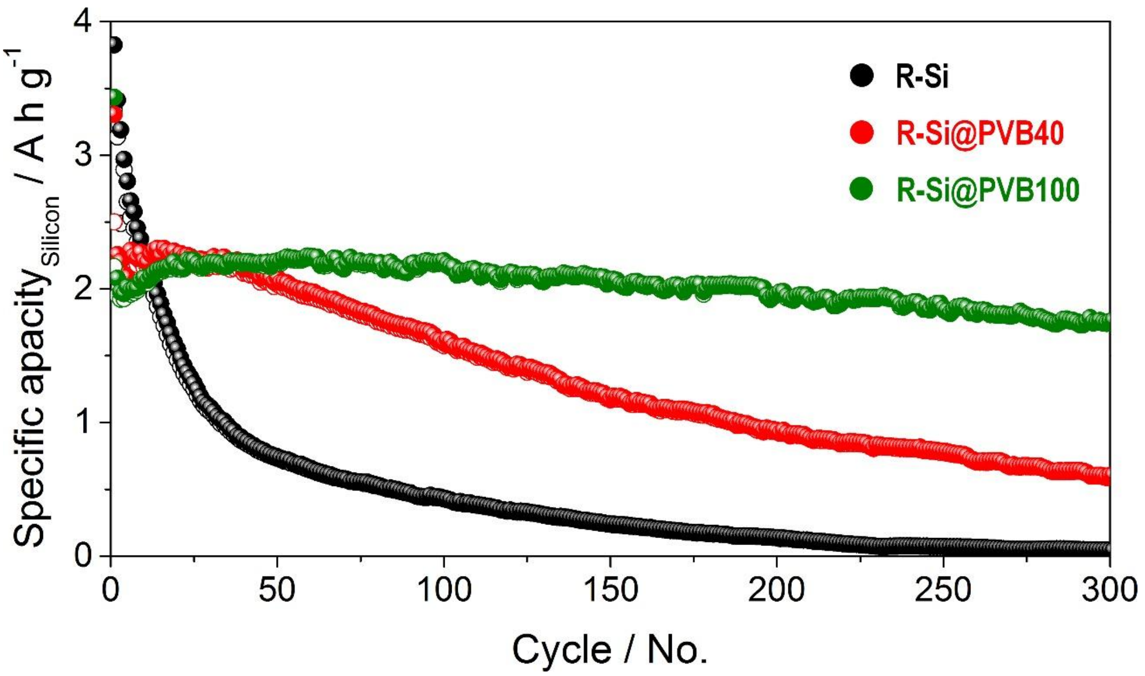


**Figure S5.** Cycle performances considering only the silicon weights of R-Si, R-Si@PVB40, and R-Si@PVB100 at a current density of 420 mA $g^{-1}$; the current density for the first cycle is 210 mA $g^{-1}$.
